# BL-YOLOv8: An Improved Road Defect Detection Model Based on YOLOv8

**DOI:** 10.3390/s23208361

**Published:** 2023-10-10

**Authors:** Xueqiu Wang, Huanbing Gao, Zemeng Jia, Zijian Li

**Affiliations:** 1 School of Information and Electrical Engineering, Shandong Jianzhu University, Jinan 250101, China; 2022085112@stu.sdjzu.edu.cn (X.W.); 2022085140@stu.sdjzu.edu.cn (Z.J.); 2021080120@stu.sdjzu.edu.cn (Z.L.); 2 Shandong Key Laboratory of Intelligent Building Technology, Jinan 250101, China

**Keywords:** BL-YOLOv8s, LSK-attention, dynamic large convolutional kernel, BiFPN

## Abstract

Road defect detection is a crucial task for promptly repairing road damage and ensuring road safety. Traditional manual detection methods are inefficient and costly. To overcome this issue, we propose an enhanced road defect detection algorithm called BL-YOLOv8, which is based on YOLOv8s. In this study, we optimized the YOLOv8s model by reconstructing its neck structure through the integration of the BiFPN concept. This optimization reduces the model’s parameters, computational load, and overall size. Furthermore, to enhance the model’s operation, we optimized the feature pyramid layer by introducing the SimSPPF module, which improves its speed. Moreover, we introduced LSK-attention, a dynamic large convolutional kernel attention mechanism, to expand the model’s receptive field and enhance the accuracy of object detection. Finally, we compared the enhanced YOLOv8 model with other existing models to validate the effectiveness of our proposed improvements. The experimental results confirmed the effective recognition of road defects by the improved YOLOv8 algorithm. In comparison to the original model, an improvement of 3.3% in average precision mAP@0.5 was observed. Moreover, a reduction of 29.92% in parameter volume and a decrease of 11.45% in computational load were achieved. This proposed approach can serve as a valuable reference for the development of automatic road defect detection methods.

## 1. Introduction

Cracks are a common issue in pavements, adversely affecting road safety and driving conditions. For transportation agencies, maintaining high-quality roads is crucial for ensuring road safety in most provinces and cities. Detecting road cracks promptly is of utmost importance in preventing road damage and ensuring traffic safety. Common methods for road defect detection include manual inspection and multifunctional road inspection vehicles. However, manual inspection is both time-consuming and labor-intensive, and it is also subject to the subjective judgment of the inspectors. In comparison, multifunctional road inspection vehicles rely on integrated sensors, such as GPS, cameras, laser profilers, and ground-penetrating radars, enabling convenient and accurate detection of road defects. In the 21st century, an increasing number of countries have introduced road-defect-detection vehicles that do not disrupt traffic during inspections. Furthermore, Roadware has developed road damage detection vehicles that can operate at night. However, the construction cost of road inspection vehicles is expensive and can reach as high as USD 500,000 [1], making them unsuitable for large-scale promotion at present. Consequently, there is a significant practical demand for the research and application of fast, efficient, and accurate crack detection technologies.

In recent years, the field of object detection has undergone significant advancements due to the rapid development of deep learning techniques. Object detection can be broadly categorized into two main approaches. The first approach is region-based two-stage detection models, which involve two distinct processes. Initially, a set of candidate regions that potentially contain objects is proposed. Subsequently, a classification network is deployed on these proposed regions to determine the object categories within each region. Popular two-stage algorithms include region-based Fast R-CNN [2], region-based Fully Convolutional Networks (R-FCN) [3], and Mask R-CNN [4], which is based on masked regions. The second approach is regression-based one-stage detection methods, which directly separate specific categories and estimate the boundaries. Although these one-stage methods offer faster processing speed compared to the two-stage approach, they tend to exhibit a slightly lower accuracy. Well-known algorithms in this category encompass the You Only Look Once [5,6] series, Single Shot MultiBox Detector (SSD) [7], and RetinaNet [8]. Presently, an increasing number of researchers are employing deep convolutional neural networks to detect and classify road cracks. For instance, Seungbo Shim et al. [9] introduced a road defect detection algorithm that combines generative adversarial networks and semi-supervised learning, achieving an average recognition accuracy of up to 81.54%. In another study, NaddafSH et al. [10] proposed the utilization of EfficientDet-D7 for the detection and classification of asphalt road images, achieving a seventh-place ranking in the 2020 IEEE Big Data Challenge. Nevertheless, it is important to note that EfficientDet-D7 suffers from drawbacks such as a large parameter size and slow detection speed, rendering it unsuitable for real-time applications. Fang Wan et al. [11]. proposed YOLO-LRDD, a lightweight algorithm for road defect detection. By using the novel backbone network Shuffle-ECANet, the algorithm reduces the model size while maintaining accuracy, making it suitable for deployment on mobile devices. Hacıefendioğlu et al. [12] used the two-stage object detection model Faster R-CNN to detect concrete road defects. They analyzed how shooting heights, distances, weather conditions, and lighting levels affect the detection performance. Arya et al. [13] used MobileNet, a lightweight network, to detect road damage images from the RDD2020 dataset in Japan, India, and Chile. They achieved an *F*1-score of 0.52. Pei et al. [14] used the Cascade R-CNN model and various data augmentation techniques. They achieved an *F*1-score of 0.635 in the Global Road Damage Detection Challenge (GRDDC 2020). However, despite the contributions of the aforementioned studies to road damage detection tasks, there is still significant room for improvement in both accuracy and detection speed. As a classic single-stage detection algorithm, the YOLO algorithm has evolved to YOLOv8, which offers significant advantages in both detection accuracy and speed. Therefore, we chose to optimize the model using the YOLOv8s framework to further enhance the algorithm’s accuracy.

## 2. YOLOv8 Network Architecture

**Figure 1:**
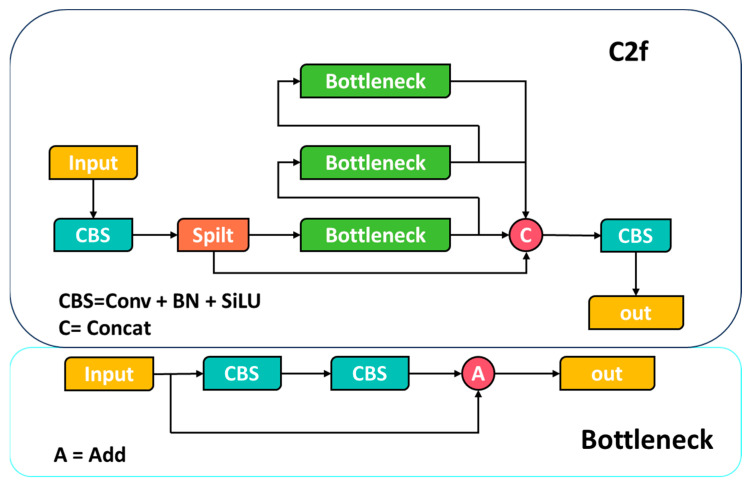
Structural diagram of the C2f module.

The YOLOv8 algorithm is a fast one-stage object detection method comprising an input segment, a backbone, a neck, and an output segment. The input segment performs mosaic data augmentation, adaptive anchor calculation, and adaptive grayscale padding on the input image. The backbone network and neck module form the central structures in the YOLOv8 network. The input image is processed by multiple Conv and C2f modules to extract feature maps at different scales. The C2f module is an improved version of the original C3 module and functions as the primary residual learning module. It incorporates the benefits of the ELAN structure in YOLOv7 [15], reducing one standard convolutional layer and making full use of the Bottleneck module to enhance the gradient branch. This approach not only preserves the lightweight characteristics but also captures more abundant gradient flow information. Figure 1 depicts the basic structure of the algorithm. The output feature maps are processed by the SPPF module, which employs pooling with varying kernel sizes to combine the feature maps, and then the results are passed to the neck layer.

The neck layer of YOLOv8 incorporates the FPN [16] + PAN [17] structure to enhance the model’s feature fusion capability. This structure combines high-level and low-level feature maps using upsampling and downsampling techniques, facilitating the transfer of semantic and localization features. Through this approach, the network becomes better equipped to fuse features from objects of varying scales, thereby enhancing its detection performance on objects at different scales.

The detection head of YOLOv8 follows the common practice of separating the classification head from the detection head. It involves loss calculation and target detection box filtering. In the loss calculation process, the TaskAlignedAssigner [18] method is used to determine positive and negative sample assignments. Positive sample selection is based on a weighted combination of classification and regression scores. The loss calculation includes two components: classification and regression, excluding the Objectness branch. The classification branch utilizes Binary Cross-Entropy (BCE) loss, while the regression branch employs the Distribution Focal Loss (DFL) [19] and CIoU loss functions. YOLOv8 prediction boxes are formed through decoupled heads, which predict classification scores and regression coordinates simultaneously. Classification scores are represented by a two-dimensional matrix, indicating the presence of an object in each pixel. Regression coordinates are represented by a four-dimensional matrix, indicating the deviation of the object’s center from each pixel. Finally, YOLOv8 employs a task-aligned assigner to compute a task alignment metric using the classification scores and regression coordinates. The task alignment metric combines the classification scores with the Intersection over Union (IoU) value, enabling the simultaneous optimization of classification and localization while suppressing low-quality prediction boxes. Intersection over Union (IoU) is a widely employed metric in object detection, serving to determine positive and negative samples as well as evaluate the distance between predicted boxes and ground truth. An object is typically classified as detected when the IoU exceeds 0.5. The specific formula is represented as Equation (1).

(1) $$IoU=\frac{│A\cap B│}{│A\cup B│}$$

## 3. Improved YOLOv8 Model

To enable the deployment of the improved model on embedded or mobile platforms, this study modifies the model structure while maintaining accuracy. The objective is to reduce the model’s parameters and computational complexity, thereby facilitating its deployment in accuracy-critical and computationally limited scenarios. Additionally, alternative methods exist to significantly reduce the model’s parameters and computational complexity, for example, by substituting the backbone of YOLOv8 with the lighter MobileNet v3 [20] network. Incorporating attention mechanisms is a commonly employed technique to enhance model accuracy as they possess fewer parameters while delivering high performance. By including attention mechanisms, the model becomes more adept at detecting targets, thereby improving detection accuracy. Following experimentation with various attention mechanisms, this study chose LSK-attention, which exhibits superior performance, to integrate into the original YOLOv8 model. The integration of BiFPN and LSK-attention reduces computational complexity while concurrently enhancing detection accuracy. Nevertheless, their inclusion leads to an augmented computational complexity of the model, consequently prolonging the inference time and reducing the FPS values during detection. To overcome this problem, the SimSPPF module was utilized to streamline the complexity of the model and reduce the inference time.

### 3.1. Feature Pyramid Optimization

**Figure 2:**
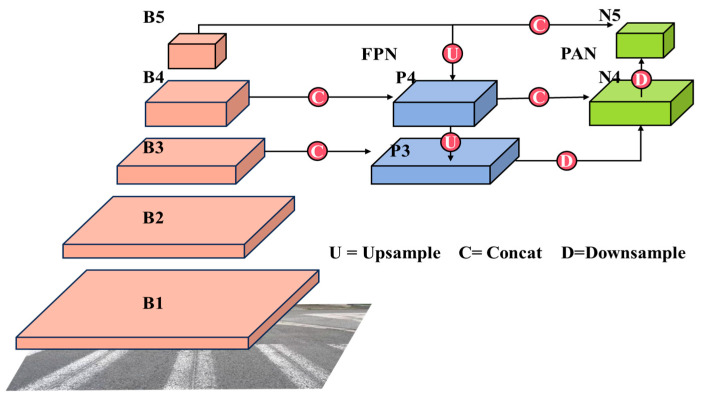
Schematic representation of the PAN-FPN (Path Aggregation Network–Feature Pyramid Network) structure in YOLOv8.

In YOLOv8, the feature maps are divided into five scales, denoted as B1-B5 for the backbone, P3-P4 for the FPN, and N4-N5 for the PAN. The original model implements the PAN-FPN structure, which is an optimized version of the traditional FPN structure. The traditional FPN structure transfers deep semantic information in a top-down manner. However, in YOLOv8, the fusion of B3-P3 and B4-P4 is performed to enhance the semantic features of the feature pyramid, potentially resulting in a partial loss of localization information. To address this, PAN-FPN introduces a bottom-up PAN structure on top of the FPN to compensate for the lost localization information. In YOLOv8, the fusion of P4-N4 and P5-N5 is employed to enhance the learning of localization features, which achieves a complementary effect. Figure 2 illustrates the specific structure diagram. Despite enriching the semantic and localization information, there is room for further improvement in the PAN-FPN structure. Firstly, the PAN-FPN structure does not adequately address large-scale feature maps, potentially overlooking valuable information and leading to a decrease in detection quality. Additionally, after upsampling and downsampling, the feature maps lose some original information, resulting in a relatively low reuse rate. Therefore, there is still potential to enhance the PAN-FPN structure.

**Figure 3:**
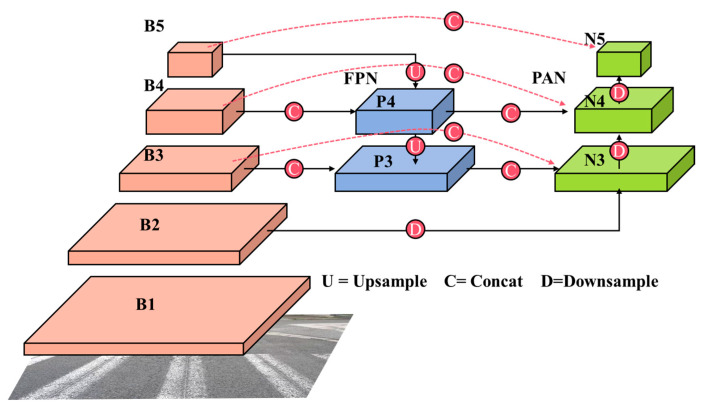
Improved neck structure.

To address the aforementioned issues more effectively, this paper introduced a reconstruction of the feature fusion component of YOLOv8, based on the concept of the Bi-directional Feature Pyramid Network (BiFPN). The BiFPN structure was initially introduced by Google in the EfficentDet [21] object detection algorithm. BiFPN enhances the semantic information of features by incorporating efficient bi-directional cross-scale connections and weighted feature fusion. In the context of road defect detection, the limited feature information extracted from smaller cracks often leads to lower detection accuracy. To overcome the challenge, consideration was given to examining the benefits of BiFPN, which expands the model’s receptive field by fully utilizing high-resolution features. The primary implementation approach is as follows: for feature maps with a single input path, no additional processing is applied due to their lower contribution. When fusing feature maps with two input paths, provided that the feature maps have the same scale, cross-level fusion requires the introduction of a new path from the backbone feature map. This enhancement improves the spatial information of the feature maps, resulting in improved detection accuracy for small targets. Figure 3 illustrates the specific structure enhancement.

### 3.2. Optimization of Spatial Pyramid Pooling

To ensure real-time performance in road defect recognition, this paper introduces the replacement of the original SPPF (Spatial Pyramid Pooling Fusion) module in the YOLOv8 model with the faster SimSPPF (Simple Spatial Pyramid Pooling Fusion) structure. The SimSPPF structure was initially introduced in YOLOv6 [22] and effectively reduces computational complexity and processing time. It achieves this by concatenating three 5x5 max pooling layers to process the input, resulting in fixed-size feature maps. These feature maps enhance the model’s receptive field and improve feature representation. Notably, SimSPPF employs the Rectified Linear Unit (ReLU) activation function, in contrast to the SPPF module, which uses the Shifted Linear Unit (SiLU) activation function. Equations (2) and (3) illustrate the specific formulas for these activation functions.

(2) ReLU: f(x)x, if x>00, if x≤0=max0,x

(3) $$Si\mathrm{LU}:\;f(x)=\frac{x^{2}}{1+e^{-x}}$$

**Figure 4:**
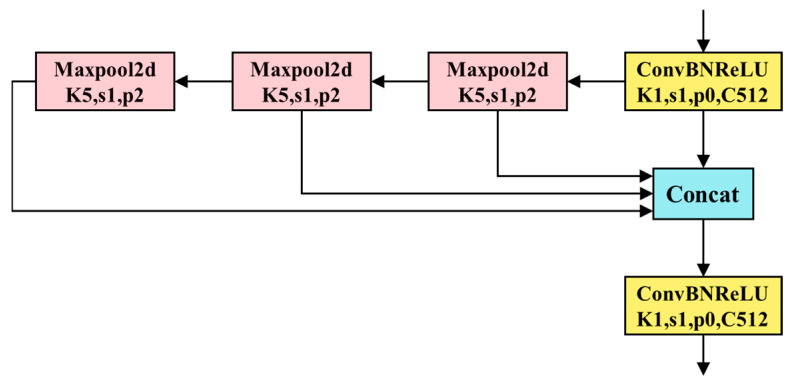
SimSPPF (Simplified Spatial Pyramid Pooling—Fast) structure diagram.

However, the use of exponential computation in the SiLU function leads to an increase in computational complexity. Consequently, replacing the SiLU function with ReLU helps address the problem of gradient vanishing and accelerates convergence. Specific experimental comparisons revealed that the execution speed of a single ConvBNReLU module is 18% faster than that of a ConvBNSiLU module. This further confirms the advantages of employing the ReLU function. Figure 4 presents the specific structure of SimSPPF.

### 3.3. Dynamic Large Convolutional Kernel Spatial Attention Mechanism

**Figure 5:**
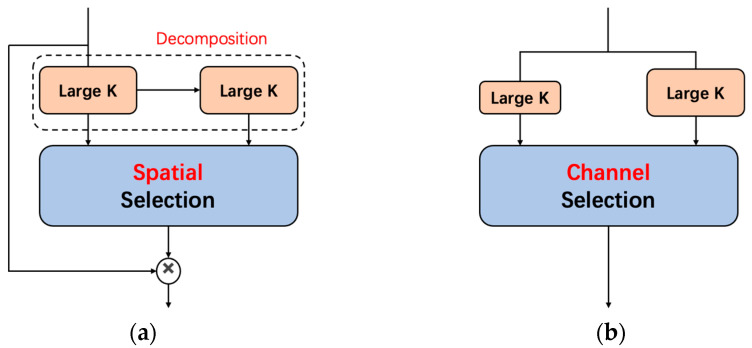
A comparison is made between the LSKNet (Large Selective Kernel Network) and SKNet (Selective Kernel Network) architectures. K: kernel; (**a**) (LSKNet); (**b**) (SKNet).

Attention mechanisms are effective in enhancing neural representations due to their simplicity and efficiency. Many excellent attention mechanisms have been developed in the field of computer vision, including channel attention mechanisms such as SE [23] modules, spatial attention mechanisms such as GeNet [24], GcNet [25], and SGE [26], and combined spatial and channel attention mechanisms such as CBAM [27] and BAM [28]. Additionally, adaptive kernel selection is a technique that effectively enhances the ability to focus on contextual regions, complementing the channel/spatial attention mechanisms. For instance, Condconv [29] and Dynamic convolution [30] employ parallel kernel adaptation to aggregate feature information from multiple convolution kernels. SKNet [31] introduces multiple convolution kernels and aggregates feature information along the channel dimension. Therefore, this paper proposes the inclusion of a dynamic large-convolution kernel selection mechanism, known as LSK-attention [32], as an output layer following the backbone. Unlike SKNet, LSK-attention adaptively aggregates feature information from large kernels in the spatial dimension, rather than utilizing the channel dimension. Figure 5 illustrates the structural comparison.

**Figure 6:**
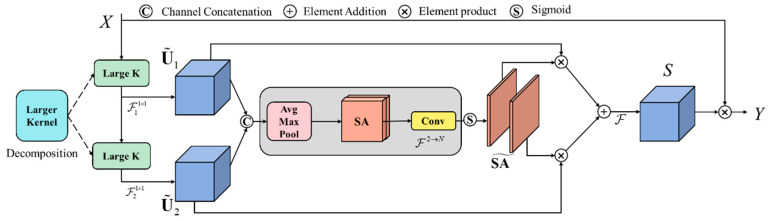
A conceptual illustration of the LSK (Large Selective Kernel) module.

The working principle of LSK-attention is depicted in Figure 6. The key advantage of LSK-attention is its utilization of multiple large convolutional kernels to generate features with a wide receptive field. These large convolutional kernels are decomposed using multiple depthwise separable convolutions, leading to a reduction in the number of model parameters. Additionally, the LSK-attention dynamically selects an appropriate convolution kernel by considering the local information of the input feature map in order to adapt to the contextual information of various target types. It also adapts its receptive field dynamically to accommodate diverse target types and backgrounds. The spatial selection mechanism is an adaptive weight allocation method that dynamically selects the most relevant feature maps from a large convolution kernel and spatially combines them. This enables enhanced focus on the most relevant spatial regions of detected targets, ultimately improving the success rate of target detection.

To dynamically select suitable spatial kernels, the input feature map is divided into multiple sub-feature maps. Subsequently, various convolutional kernels of different sizes are applied to each sub-feature map, resulting in the generation of multiple output feature maps. Afterward, these sub-output feature maps are concatenated, as depicted in Equation (4). This concatenation results in an output feature map with increased channel dimensions.

(4) $$\tilde{U}=[{\tilde{U}}_{1};\ldots ;{\tilde{U}}_{i}]$$

Subsequently, the concatenated feature map undergoes average pooling and max pooling operations along the channel dimension to extract spatial relationship descriptors, namely SA*_avg_* and SA*_max_*. The specific operation is illustrated in Equation (5).

(5) $$SA_{avg}=P_{avg}(\tilde{U}),SA_{max}=P_{max}(\tilde{U}),$$

Subsequently, following the concatenation of SA*_avg_* and SA*_max_*, convolutional layers are utilized to transform them into spatial attention maps, ensuring they possess the same number of depth convolutions N. This conversion is mathematically expressed by Equation (6).

(6) $$\hat{SA}=F^{2\to N}([SA_{avg};SA_{max}])$$

By applying the sigmoid activation function to each spatial attention map, the spatial selection weights for each depth convolution are obtained. The weighted depth convolution feature maps are subsequently acquired by element-wise multiplication of the weights and the corresponding depth convolutions. Ultimately, a convolutional layer is employed to fuse these feature maps and produce the final attention feature. This process is mathematically demonstrated by Equations (7) and (8).

(7) $$\tilde{SA_{i}}=Sigmoid(\hat{SA_{i}})$$

(8) $$S=F(\overset{N}{\underset{i=1}{\sum }}({\tilde{SA}}_{i}\cdot {\tilde{U}}_{i}))$$

### 3.4. Network Structure and Parameters

Table 1 presents the pertinent information to offer readers a comprehensive understanding of the network structure and detailed parameters of BL-YOLOv8s.

**Figure 7:**
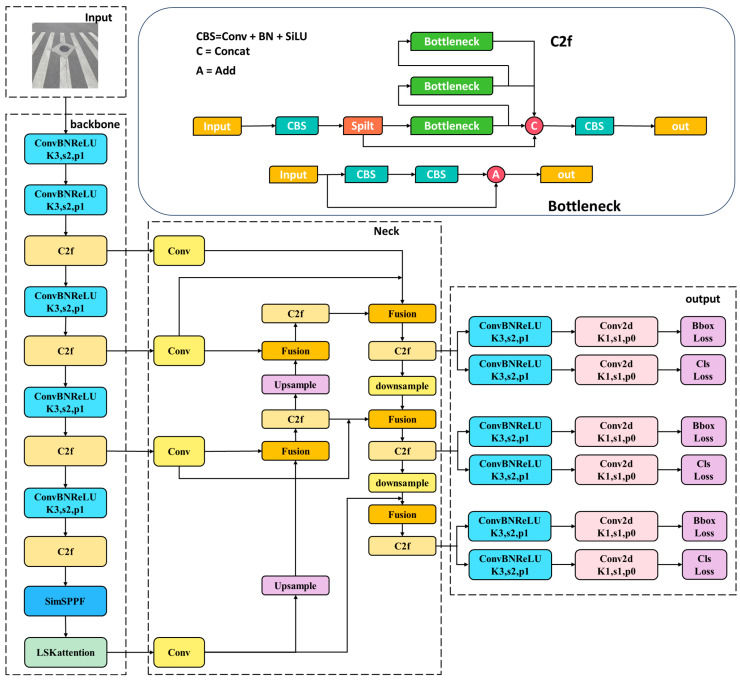
Improved structure of YOLOv8s.

The structure of the final implemented YOLOv8 is shown in Figure 7.

## 4. Results and Analysis

### 4.1. Experimental Environment

In order to verify the efficacy of the proposed approach, an experimental platform was set up employing Ubuntu 18.04 as the operating system and PyTorch as the deep learning framework. YOLOv8s was employed as the baseline network model. The specific configuration of the experimental environment is elaborated in Table 2.

Consistent hyperparameters were applied throughout the training process across all experiments. Table 3 displays the precise hyperparameters employed during the training process.

### 4.2. Dataset and Evaluation Metrics

**Figure 8:**
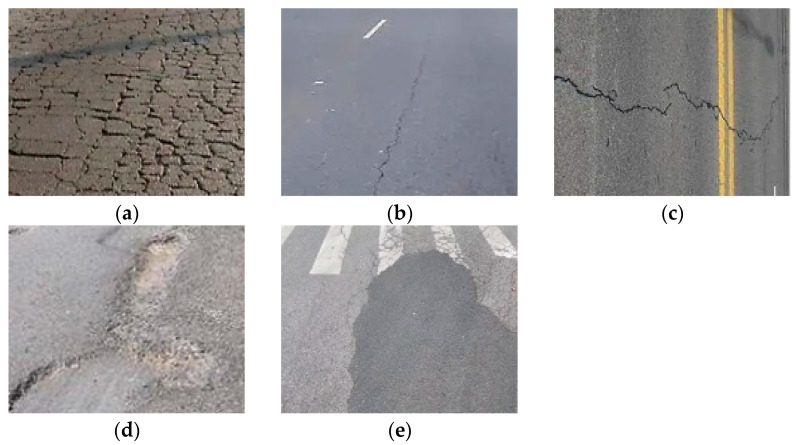
Examples of different types of defects: (**a**) grid cracks D20; (**b**) longitudinal cracks D00; (**c**) transverse cracks D10; (**d**) potholes D40; and (**e**) road repair.

In this study, we utilized the open-source dataset RDD2022 [13], consisting of road images from various countries. For experimental validation, a total of 4398 road images from China were selected. These images include 2401 captured by a drone and 1977 captured by a vehicle-mounted camera. Five types of road defects are considered in this study: longitudinal cracks (D00), transverse cracks (D10), grid cracks (D20), potholes (D40), and road repairs (Figure 8). Due to the absence of the five types of road defects considered in this study in certain photos of the training set, it becomes necessary to process and filter the dataset to exclude images that do not depict the detection targets of this study. Following analysis and processing, the dataset comprises 4373 images depicting the detection targets of this study. The quantity of different types of road defects in the dataset is presented in Table 4. The images are divided into training, validation, and test sets in an 8:1:1 ratio.

To provide an objective assessment of the performance of road defect detection models, the evaluation metrics employed encompass GFLOPS (giga floating-point operations per second), which quantifies the execution time of the network model in terms of billions of floating-point operations per second. The parameters, which assess the size and complexity of the model. FPS (Frames Per Second), which gauges the detection speed of the model in frames transmitted per second. *mAP* (mean average precision), utilized to evaluate the model’s accuracy, is computed using Equation (9). The *F*1-score, which is a weighted average of precision and recall, serves as a measure of the model’s overall performance and stability. The calculation formula for the *F*1-score is provided in Equation (10).

(9) $$mAP=\frac{\sum P_{A}}{N}$$

(10) $$F1-\mathrm{score}=2*\frac{Precision*Recall}{Precision+Recall}$$

In Equation (9), N represents the summary of categories. *P_A_* is the area under the curve formed by plotting recall on the x-axis against precision on the y-axis. mAP@0.5 represents the average precision at a threshold of 0.5. Equation (10) demonstrates how precision represents the model’s capability to differentiate negative samples. A higher precision value indicates a more effective distinction among negative samples. Recall showcases the model’s capability to identify positive samples. A higher recall value indicates a more accurate identification of positive samples. The *F*1-score represents a combination of precision and recall, with a higher value indicating a model with stronger performance and robustness.

### 4.3. Comparison of Different Spatial Pyramid Pooling Effects

The impact of various spatial pyramid pooling layers, namely SPP, SPPF, SimSPPF, ASPP, SPPCSPC, and SPPFCSPC, on model size and accuracy is compared in Table 5. Among them, SPPCSPC achieves the highest accuracy, but it also significantly increases the model’s parameter count and computational complexity, resulting in a longer processing time compared to other modules. However, the SimSPPF module enhances the model’s speed without increasing the computational and parameter count. It only exhibits a 0.7% difference in accuracy compared to SPPCSPC but is four times faster in computation speed and 1.2 times faster than the original SPPF module in YOLOv8. For this reason, SimSPPF is adopted in subsequent research because it improves the accuracy and speed of the object detection model with minimal modifications to the network.

### 4.4. Comparison of Different Attention Mechanism Effects

The impact of integrating different attention modules at the end of the backbone on the model’s detection accuracy is presented in Table 6. This set of experiments is conducted on the YOLOv8s model, with the neck part reconstructed as illustrated in Figure 4. From Table 4, it is evident that incorporating various attention modules leads to a slight increase in the computational and parameter count of the model, while the MAP@0.5 value of the model shows some variation. Adding the channel attention module alone results in a modest enhancement in mAP@0.5, although in certain cases, a decrease in accuracy is observed (e.g., CA [33]). However, when both channel attention and spatial attention are combined within the CBAM module in YOLOv8, the network’s accuracy improves even further compared to YOLOv8 with only channel attention, with an improvement of up to 0.9%. Consequently, the inclusion of spatial attention contributes to a higher accuracy in the network. Additionally, the introduced spatial attention mechanism, LSK-attention, achieves an impressive model accuracy of 90.5%, with a remarkable improvement rate of 1.2%. Moreover, it demonstrates faster detection speed compared to the model with the CBAM module incorporated.

### 4.5. Ablation Experiment

**Figure 9:**
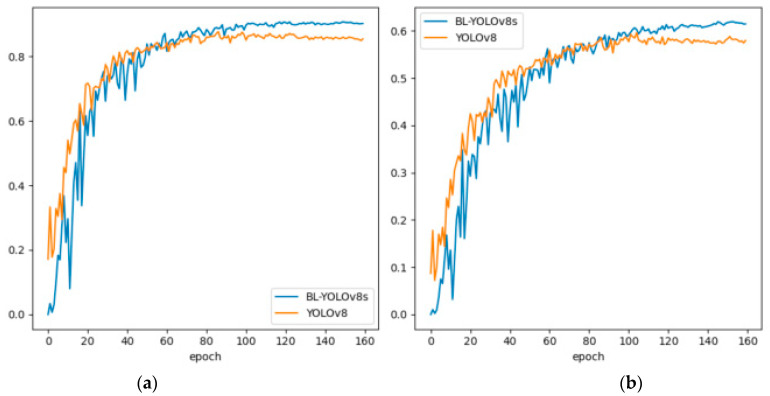
A comparison was conducted to evaluate the mAP values of the BL-YOLOv8 model against the original. (**a**) Comparison of mAP@0.5, and (**b**) comparison of mAP@0.5:0.95.

Based on the data presented in Table 7, reconstructing the neck part of YOLOv8s with the BiFPN concept led to a 33.78% reduction in the parameter count and a 12.5% decrease in computational complexity. Furthermore, the model’s accuracy (MAP@0.5) improved by 1.9%. Replacing SPPF with SimSPPF had minimal effects on the model’s accuracy, parameter count, and computational complexity. However, it did increase the detection speed of the model by 2 FPS. Introducing the LSK-attention module resulted in a slight increase in the parameter count and computational complexity, but it yielded an additional improvement of 1.3 percentage points in accuracy (mAP@0.5). Combining BiFPN with the SimSPPF module enhanced the model’s accuracy while reducing the computational and parameter count of the network. Overall, the improved version of YOLOv8, incorporating BiFPN, SimSPPF, and LSK-attention modules, surpassed the original YOLOv8 model in terms of detection accuracy, computational complexity, and parameter count. On the same dataset, the enhanced YOLOv8 model achieved a 3.3% improvement in mAP@0.5, a 29.92% reduction in parameter count, and an 11.45% reduction in computational complexity. For a comparison of accuracy between the original and improved models, please refer to Figure 9.

The analysis of Figure 9 indicates that the BL-YOLOv8s model, as proposed, exhibits significantly higher values for mAP@0.5 and mAP@0.5:0.95 in comparison to the original YOLOv8 model.

### 4.6. Interpretability Experiment

**Figure 10:**
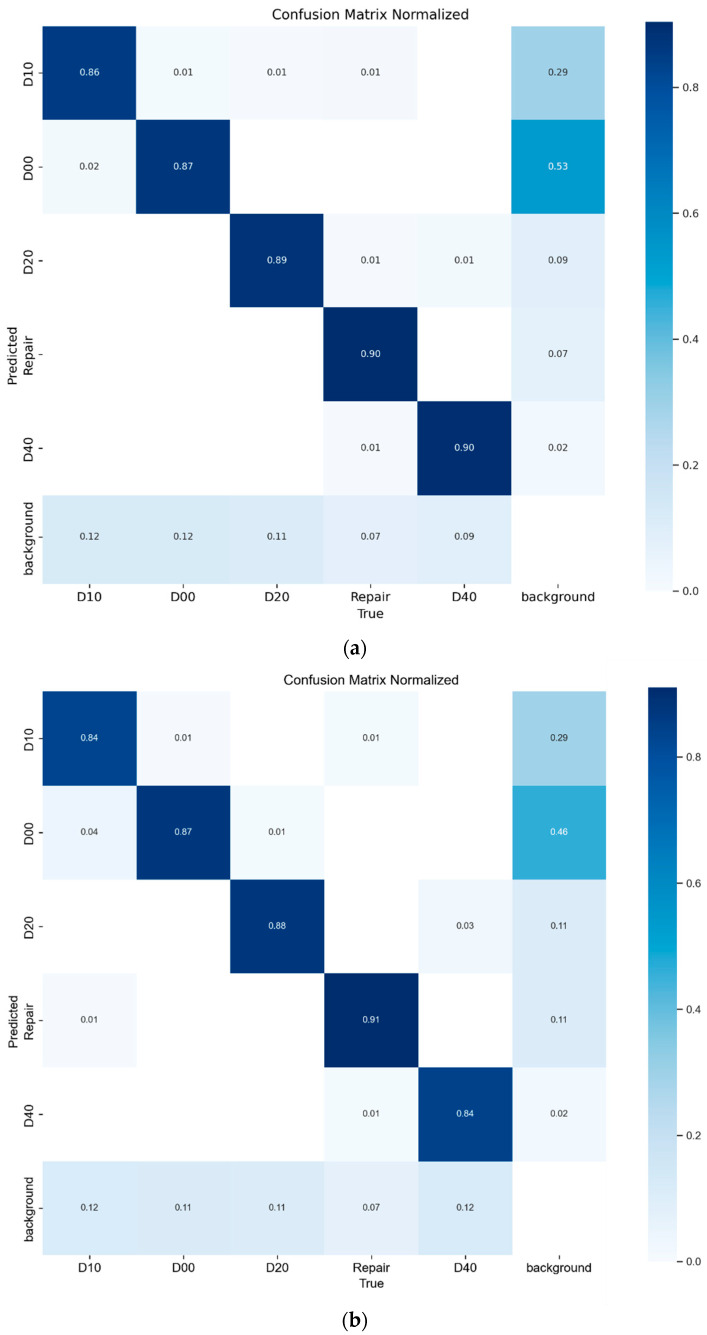
Confusion matrix of the BL-YOLOv8 model and YOLOv8 model. (**a**) Confusion matrix of BL-YOLOv8. (**b**) Confusion matrix of YOLOv8.

Deep learning models are often regarded as black-box models, making it challenging to interpret their decision-making and reasoning processes, despite their outstanding performance in different tasks. Understanding the interpretability of deep learning models is crucial in vital domains like medical diagnosis, autonomous driving, and financial prediction. Thoroughly exploring the interpretability of deep learning models is essential for developing an intuitive understanding of their performance. In this experiment, we selected the BL-YOLOv8 model and the YOLOv8 model as the models for verification. We thoroughly examined their performance by analyzing the confusion matrices of both models. Figure 10 displays the confusion matrices of the two models.

The confusion matrices indicate that both models exhibit high rates of false negatives (i.e., misclassifying targets as background categories) and false positives. Detailed analysis demonstrates the strong performance of both models in recognizing D20 (grid cracks) and repair (road repair). The original YOLOv8 model exhibited subpar recognition performance for D10 (transverse cracks) and D40 (potholes), achieving an accuracy rate of only 84%. In contrast, the BL-YOLOv8 model proposed in this paper significantly enhanced the recognition accuracy of both D10 and D40, with improvements of 2% and 6%, respectively.

### 4.7. Self-Built Data Performance Experiments

**Figure 11:**
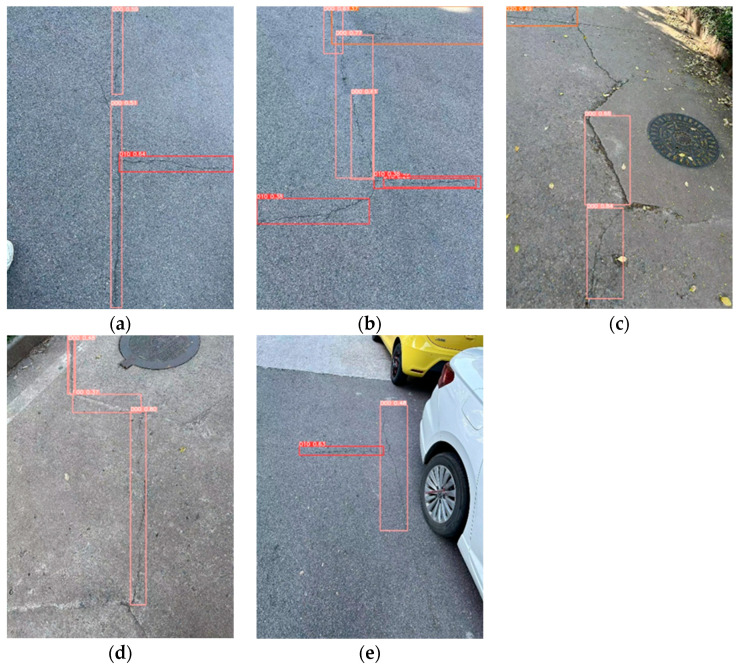
(**a**–**e**) Detection results from self-collected data.

To enhance the validation of the BL-YOLOv8 model’s universality, specific performance tests were conducted on the model using real road images. Shandong Jianzhu University was selected as the shooting location with photography taking place at 15:00 in the afternoon. Images were captured using an iPhone 14pro device. The camera settings included an ISO value of 80 and an aperture of f/1.78. The captured images had dimensions of 3024 × 4032 pixels. Various sets of sample images were captured to assess the performance of the model. The specific detection results are presented in Figure 11.

The detection results displayed in Figure 11 demonstrate the effectiveness of the BL-YOLOv8 model by successfully identifying the majority of road defects. This also highlights the model’s strong generalization capabilities and robustness. Nevertheless, certain instances of missed and false detections persist. For instance, in Figure 11c situated above the image, a longitudinal crack went undetected. This can be ascribed to the unclear boundaries of road cracks in certain captured images, causing the detection bounding boxes to only partially align with the crack boundaries, as depicted in Figure 11e.

### 4.8. Comparison of Performance of Different Models

To assess the performance of the enhanced model, this study conducted comparative experiments between the enhanced model and various widely used object detection models. The chosen models encompass both two-stage anchor-based approaches, including Faster R-CNN, and one-stage anchor-based approaches like SSD, YOLOv3, YOLOv5, and YOLOv7. Additionally, one-stage anchor-free models such as YOLOv6 and YOLOX [36] were examined, along with the Guo [37]-improved YOLOv5 model and the Pham V [38]-improved YOLOv7 model. The experiments were carried out on the same dataset and under identical experimental conditions. For comparative analysis, two road condition images captured by unmanned aerial vehicles (UAVs) and one road condition photo taken by an onboard camera from the RDD2022 dataset were chosen for detection and recognition. The figure illustrates the detection results, with detection boxes of various colors that indicate distinct defect categories, accompanied by annotations for labeling various types of defects. For instance, D10 signifies transverse cracks, D20 corresponds to grid cracks, D00 denotes longitudinal cracks, D40 symbolizes potholes, and repair indicates road repairs.

**Figure 12:**
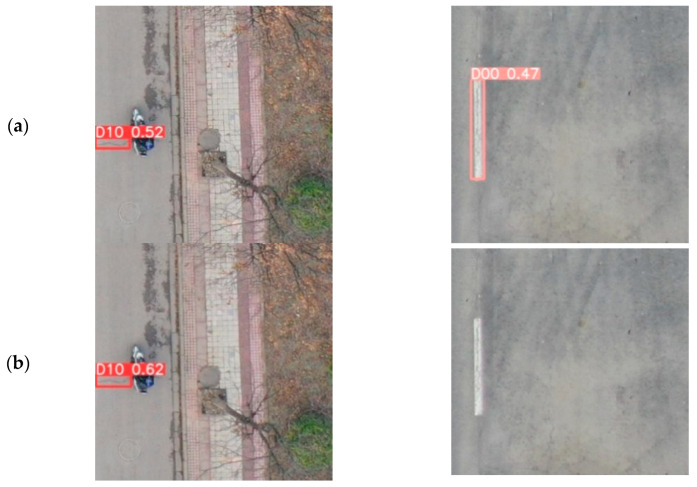

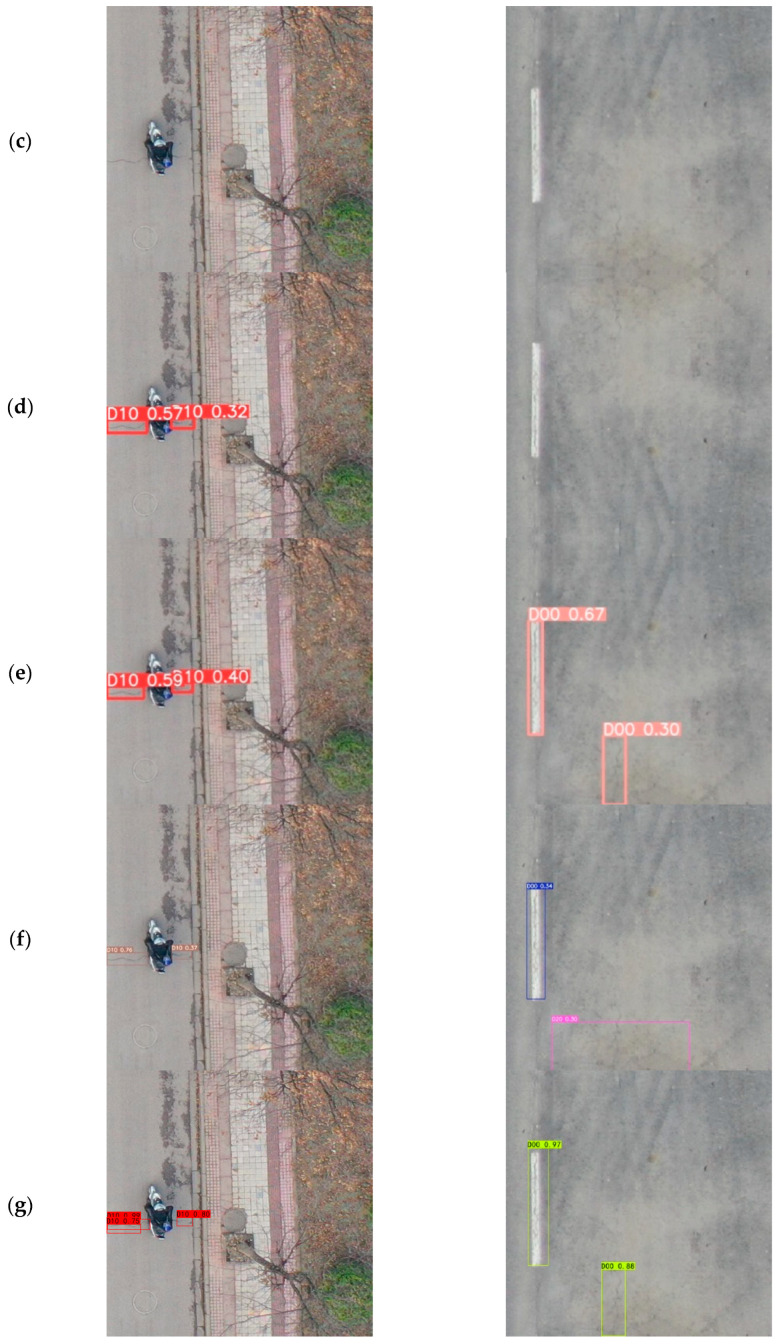

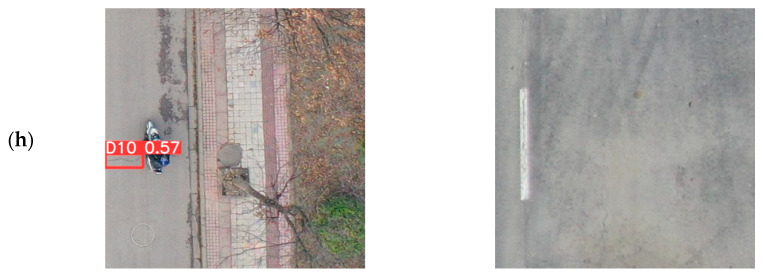
Visualization results of comparative experiments for seven models in drone-captured scenes: (**a**) YOLOv8s; (**b**) YOLOv5s; (**c**) SSD; (**d**) YOLOv6s; (**e**) BL-YOLOv8; (**f**) YOLOv7-tiny; (**g**) Faster-RCNN; (**h**) YOLOv3-tiny.

In Experiment 1, which involved drone-captured images, the detection results of various models are displayed in Figure 12. Models such as SSD, YOLOv5s, YOLOv6s, YOLOv3-tiny, and YOLOv8 demonstrate different levels of missed detections, with SSD being the most severely affected, even leading to cases where all targets are missed. Despite successfully detecting all targets, the YOLOv7-tiny model also exhibits false positive detections. While the Faster-RCNN model does not suffer from false positive or missed detection issues, its predicted bounding boxes are inaccurate and may even overlap. On the other hand, the proposed BL-YOLOv8 model exhibits outstanding performance in this particular scenario, with minimal missed detections or false positives, and its predicted bounding boxes are more accurate compared to the other models.

**Figure 13:**
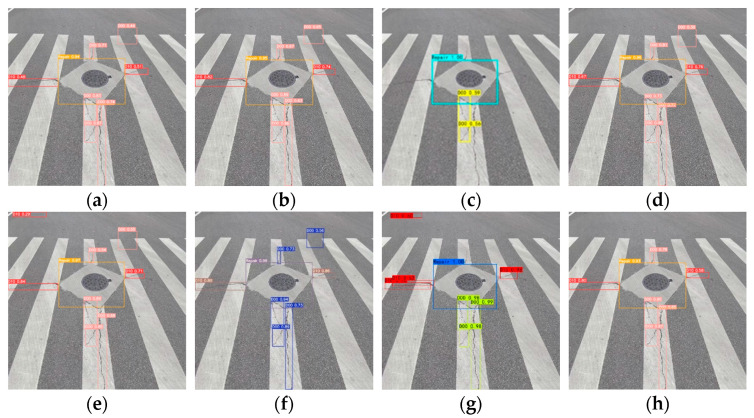
Visualization results of comparative experiments for seven models conducted in scenes captured by a vehicle-mounted camera): (**a**) YOLOv8s; (**b**) YOLOv5s; (**c**) SSD; (**d**) YOLOv6s; (**e**) BL-YOLOv8s; (**f**) YOLOv7-tiny; (**g**) Faster-RCNN; and (**h**) YOLOv3-tiny.

The detection results of the different models in Experiment 2 (capturing images from onboard cameras) can be seen in Figure 13. YOLOv8s, SSD, and Faster-RCNN models exhibited recurrent instances of missed or falsely detected targets, whereas YOLOv5, YOLOv6s, YOLOv7-tiny, YOLOv3-tiny, and our proposed BL-YOLOv8s model effectively identified all targets. Conversely, YOLOv3-tiny encountered false detection problems. BL-YOLOv8 outperformed the preceding three models by producing more precise bounding boxes during target detection. The comprehensive results from both experiments reveal the superior detection accuracy, lower missing detection rate, and decreased false detection rate of the BL-YOLOv8s model compared to other models in various scenarios. These findings serve as evidence for the outstanding performance of our model across diverse scenarios, significantly enhancing target detection.

As shown in Table 8, the BL-YOLOv8 model attained a high Map@50 accuracy of 90.7% and an *F*1-score of 0.87, markedly higher compared to other object detection models. These results indicate the superior performance and stability of the BL-YOLOv8 model. Moreover, apart from enhancing the mAP@50 and *F*1-score, the model witnesses reductions of 29.92% and 11.45%, respectively, when compared to the original YOLOv8 model in terms of both parameter and computational overhead.

## 5. Discussion

The accuracy in detecting road defects was improved in the proposed BL-YOLO model compared to the original YOLOv8 model. Nonetheless, certain road defects remain undetected, potentially attributed to the following factors. The first reason relates to the characteristics of the images. Road defects may vary under different weather conditions and road types. Moreover, blurred road images can arise from small cracks and long shooting distances, leading to increased detection challenges. The second reason is the complexity of road conditions, where acquired images encompass not only road information but also interfering factors like pedestrians, vehicles, and obstacles. Consequently, these interfering factors pose challenges to the detection of road cracks. The third reason pertains to the BL-YOLOv8 model. Despite the enhanced ability of the BL-YOLOv8 model to detect small targets compared to the original YOLOv8 model, it still encounters limitations in detecting small targets that closely resemble the surrounding environment.

Furthermore, the proposed BL-YOLOv8 model exhibits substantial improvements in accuracy, mAP@0.5, parameter quantity, and computational complexity when compared to the original YOLOv8 model. Nevertheless, these enhancements are accompanied by increased inference time. While the adoption of the BIFPN structure can decrease the model’s parameter quantity and computational complexity, the integration of multi-level features across scales in this structure inevitably heightens the computational complexity compared to the original FPN-PAN structure. The experimental results revealed that the introduction of the FPN structure prolongs inference time and reduces the model’s detection speed. To tackle this problem, the SimSPPF structure was introduced to replace the original SPPF structure. Through the implementation of a lower computational cost ReLU activation function, SimSPPF aids in mitigating the model’s computational complexity. Experimental findings indicate that the introduction of the SimSPPF structure can enhance the model’s detection speed. Furthermore, experimental results suggest that the incorporation of attention mechanisms can effectively enhance detection performance. Nonetheless, this approach comes with the drawback of augmenting computational and parameter complexity, as well as inference time, rendering it more demanding for real-time detection tasks.

The incorporation of the BiFPN structure enhances the feasibility of subsequent model updates and fine-tuning. For instance, it enables the fusion of features across multiple layers at varying levels. The LSK-attention is exclusively integrated after the backbone of the YOLOv8 model, resulting in minimal disruption to the model’s original structure, thereby preserving unaffected future updates and fine-tuning. Achieving precise detection of road defects is of utmost importance for intelligent road maintenance and ensuring road safety. To facilitate the application of this model in road intelligent maintenance equipment, the BL-YOLOv8 model reduces both the number of parameters and computational complexity in comparison to the original YOLOv8 model. This offers the potential to deploy it on cost-effective embedded devices or mobile devices. Furthermore, this model relies primarily on image data. When integrated with a camera on a development board, it can establish a comprehensive visual recognition system, resulting in a significant reduction in deployment costs.

## 6. Conclusions

Accurately detecting road defects is essential for implementing intelligent road maintenance. This paper presents a road defect detection model based on an enhanced version of YOLOv8s. In the proposed approach, the Neck structure of the original model is reconstructed using the BiFPN method to reduce the model size and enhance its feature fusion capability. Subsequently, the SimSPPF module is employed to optimize the spatial pyramid pooling layer and enhance the model’s detection speed. Finally, the LSK attention mechanism, which employs dynamic large convolutional kernels, is introduced to enhance the model’s detection accuracy. Experimental results demonstrate the proposed model’s effective performance in detecting road defects from images captured by drones and vehicle-mounted cameras. This provides evidence that the model is suitable for various detection scenarios. BL-YOLOv8 outperforms other mainstream object detection models (e.g., Faster R-CNN, SDD, YOLOv3-tiny, YOLOv5s, YOLOv6s, and YOLOv7-tiny) by achieving detection accuracy improvements of 17.5%, 18%, 14.6%, 5.5%, 5.2%, 2.4%, and 3.3%, respectively. Additionally, the BL-YOLOv8s model enhances detection accuracy while reducing computational and parameter demands. In comparison to the original YOLOv8s model, the parameter size is reduced by 29.92%, and the computational load is reduced by 11.45%. This makes the proposed model suitable for scenarios with memory and computation constraints, such as embedded devices. In our future research, we intend to gather diverse road images from multiple cities, including highway and urban arterial road images. This will contribute to improving the model’s generalization and stability. Furthermore, we aim to collect additional data, including wavelength, vibration, and other parameters, to integrate with the image information. This integration will result in further enhancements to the model’s reliability.

## Figures and Tables

**Table 1:** Network structure and parameters of BL-YOLOv8s.

Layers	From	N	Params	Module	Arguments
0	−1	1	928	Conv	[3, 32, 3, 2]
1	−1	1	18,560	Conv	[32, 64, 3, 2]
2	−1	1	29,056	C2f	[64, 64, 1, True]
3	−1	1	73,984	Conv	[64, 128, 3, 2]
4	−1	2	197,632	C2f	[128, 128, 2, True]
5	−1	1	295,424	Conv	[128, 256, 3, 2]
6	−1	2	788,480	C2f	[256, 256, 2, True]
7	−1	1	1,180,672	Conv	[256, 512, 3, 2]
8	−1	1	1,838,080	C2f	[512, 512, 1, True]
9	−1	1	656,896	SimSPFF	[512, 512, 5]
10	−1	1	433,350	LSK	[512]
11	4	1	16,640	Conv	[128, 128]
12	6	1	33,024	Conv	[256, 128]
13	10	1	65,792	Conv	[512, 128]
14	−1	1	0	Upsample	[None, 2, ’nearest’]
15	[−1, 12]	1	2	Fusion	[[128, 128], ’bifpn’]
16	−1	1	115,456	C2f	[128, 128, 1]
17	−1	1	0	Upsample	[None, 2, ’nearest’]
18	[−1, 11]	1	2	Fusion	[[128, 128], ’bifpn’]
19	−1	1	115,456	C2f	[128, 128, 1]
20	2	1	73,984	Conv	[64, 128, 3, 2]
21	[−1, 11, 19]	1	3	Fusion	[[128, 128, 128], ’bifpn’]
22	−1	1	115,456	C2f	[128, 128, 1]
23	−1	1	147,712	Conv	[128, 128, 3, 2]
24	[−1, 12, 16]	1	3	Fusion	[[128, 128, 128], ’bifpn’]
25	−1	1	115,456	C2f	[128, 128, 1]
26	−1	1	147,712	Conv	[128, 128, 3, 2]
27	[−1, 13]	1	2	Fusion	[[128, 128], ’bifpn’]
28	−1	1	115,456	C2f	[128, 128, 1]
29	[22, 25, 28]	1	1,262,272	Detect	[80, [128, 128, 128]]
summary (fused): 204 layers, 7829,394 parameters, 7829,378 gradients, 25.5 GFLOPs					

**Table 2:** Configuration and training environment.

Environmental Parameter	Value
Operating system	Ubuntu18.04
Deep learning framework	Pytorch
programming language	Python3.8
CPU	Intel(R) Xeon(R) Platinum 8255C
GPU	RTX 3090 (24 GB)
RAM	30 GB

**Table 3:** Hyperparametric configuration.

Hyperparameters	Value
Learning Rate	0.01
Image Size	640 × 640
Momentum	0.937
Optimizer	SGD
Batch Size	64
Epoch	160
Weight Decay	0.0005

**Table 4:** Types and quantities of defects in the dataset.

Pavement Distress	Distress Class	Quantity
grid cracks	D20	756
longitudinal cracks	D00	3270
transverse cracks	D10	1895
potholes	D40	255
road repair	Repair	821

**Table 5:** Performance table of different spatial pyramid pooling layers under multiple metrics.

Models	mAP@0.5/%	Para (M)	GFLOPs	Time (ms)
Yolov8s + SPP	86.9	11.16	28.8	16.40
Yolov8s + SPPF	87.4	11.16	28.8	6.93
Yolov8s + SimSPPF	87.4	11.16	28.8	5.73
Yolov8s + ASPP	86.8	14.44	31.2	11.12
Yolov8s + SPPCSPC	88.1	17.59	34.0	29.72

**Table 6:** Performance table of different attention modules.

Models	mAP@0.5/%	Para (M)	GFLOPs	FPS
YOLOv8s + BiFPN	89.3	7.39	25.2	108
+SE	89.3	13.82	30.1	83
+Biform [34]	89.4	8.42	25.2	98
+CBAM	90.2	7.39	25.1	91
+EMA [35]	89.3	7.40	25.3	98
+CA	89.0	7.42	25.1	111
+LSK-attention	90.5	7.83	25.7	103

**Table 7:** Ablation experiments with the modules.

BiFPN	SimSPPF	LSK-Net	mAP@0.5/%	Para (M)	GFLOPs	FPS
			87.4	11.16	28.8	128
√			89.3	7.39	25.2	115
	√		87.4	11.16	28.8	130
		√	88.7	11.58	29.0	120
√	√		89.5	7.39	25.2	117
√	√	√	90.7	7.82	25.5	98

**Table 8:** Performance comparison of different models in detection.

Models	mAP@0.5/%	Para (M)	GFLOPs	FPS	*F*1-Score
Faster R-CNN	73.2	28.31	940.97	11	0.60
SSD	72.7	26.28	62.74	76	0.61
YOLOv3-tiny	76.1	8.68	13.0	222	0.71
YOLOv5s	85.2	7.02	15.8	156	0.83
YOLOv6s	85.5	16.29	44.0	108	0.81
YOLOv5-MobileNetv3 [37]	87.1	7.39	9.9	82	0.84
YOLOX	89.0	5.06	15.4	77	0.86
Efficientdet [10]	49.8	3.87	5.2	27	0.46
YOLOv7-CA [38]	87.0	6.02	13.1	138	0.82
YOLOv7-tiny	88.3	6.02	13.2	144	0.84
YOLOv8s	87.4	11.16	28.8	120	0.83
BL-YOLO8s	90.7	7.82	25.5	98	0.87

## Data Availability

The dataset is sourced from RDD2022, and it can be downloaded from the following website. https://crddc2022.sekilab.global/data/ (accessed on 26 September 2022).
